# Acute Effects of Stretching Exercises on Posterior Chain: Analysis of Shear Modulus by Elastography SSI

**DOI:** 10.1155/2023/5582277

**Published:** 2023-12-28

**Authors:** Maria Clara Albuquerque Brandão, Gabriela de Carvalho Teixeira, Liliam Fernandes de Oliveira

**Affiliations:** Laboratório de Biomecânica, Programa de Engenharia Biomédica—COPPE, Federal University of Rio de Janeiro (UFRJ), Rio de Janeiro, Brazil

## Abstract

The posterior chain muscles of the lower limb include the hamstrings and triceps surae, along with the Achilles tendon. This study aimed to investigate the acute effects of static stretching exercises commonly used in clinical and training settings on the shear modulus (*µ*) of these muscles and tendon using Supersonic Shear-Wave Imaging (SSI) elastography. Fifteen healthy adults participated in the study, performing stretching exercises for hamstrings and triceps surae. Shear modulus and joint range of motion (ROM) were measured before and after the stretching protocols. The hip and ankle mean ROM significantly increased by 19.27% and 24.10%, respectively. However, the stretching protocol did not significantly alter in *µ* of the hamstrings, the gastrocnemius muscles, and the Achilles tendon. *K*-means clustering analysis identified a group where the subjects with lower initial ROM showed higher amplitude gains and a significant decrease in the semimembranosus stiffness after stretching. These findings suggest that the stretching protocol was effective in improving joint mobility but not sufficient to elicit immediate mechanical changes in muscle and tendon stiffness. Neural adaptations and nonmuscular structures might contribute to increased ROM. The study highlights the importance of considering individual initial ROM and subsequent responses when evaluating the effects of stretching exercises on muscle and tendon properties.

## 1. Introduction

The hamstrings muscle group, composed of semitendinosus (ST), semimembranosus (SM), and biceps femoris (BF) [1], and the triceps surae muscle group comprising the medial gastrocnemius (MG), lateral gastrocnemius (LG), soleus muscles, and Achilles tendon (AT) [2] collectively form the posterior chain of the lower limbs. Most of these muscles are biarticular, acting as hip extensors, knee flexors, and plantar flexors, and are crucial for performing daily tasks that involve high loads and large ranges of motion (ROM) [3].

Stretching programs targeting these muscle groups are commonly recommended to improve flexibility and enhance sports performance and quality of life by increasing ROM [4]. However, there is limited knowledge about the precise neural and mechanical factors contributing to this increased ROM, including acute adaptations in stretch tolerance, sensory perception, mechanical alterations, and tissue properties such as stiffness [5, 6]. Gains of the maximum joint ROM are usually related to the stretching protocol efficacy. However, the various factors that contribute to increased ROM, including acute neural adaptations such as stretch tolerance [7, 8] and alterations in sensory sensations like stretch perception [9], acute mechanical alterations, reductions in the passive torque curve [10], and transient alterations in the mechanical properties of the tissue [11], particularly its stiffness.

Efforts to understand these mechanical adaptations in vivo have led to the utilization of shear wave elastography (SWE), specifically the Supersonic Shear-Wave Imaging (SSI) type. This imaging technique quantifies the tissue shear modulus (*µ*) noninvasively and in real time, with its value being indicative of medium stiffness [12]. The technique is based on the emission of high-intensity acoustic radiation forces, focused on different depths of the tissue, generating transverse waves within the tissue [13]. Simultaneously, other elements of the transducer calculate the propagation speed of these shear waves (*c*_*s*_) in an ultra-fast way [12, 13]. Assuming isotropic and purely elastic tissue and considering the density of the biological tissue (*ρ*) of 1010 kg/m^3^, the shear modulus is estimated as *μ*=*ρ*.*c*_*s*_^2^.

Several studies have established the reliability of this technique for evaluating muscles and tendons in the lower limb. The reliability of the gastrocnemius in longitudinal images obtained an ICC range of 0.73 to 0.96 [14], and a similar range (ICC: 0.85) was also verified for the relaxed long head of the BF [15]. Another study indicates that elastography is effective in assessing in *µ* of lower limb muscles (MG, LG, BF, ST, and SM), especially at rest, with higher reproducibility [16]. For the AT, the measurement repeatability is moderate [17].

With this method, some studies have investigated the acute effect of stretching on the hamstrings and reported an increase in ROM and a significant reduction in *µ* of biceps femoris long head (BF-lh), SM, and ST after a static stretching with an isokinetic dynamometer, with a duration between 2.5 minutes and 7.5 minutes [18–20]. For the triceps surae, the results are controversial. A significant reduction in the stiffness of the gastrocnemius was observed after 5 minutes of static stretching using a stretching board [21] or a standing wall stretch [22], while others reported no significant changes in *µ* for gastrocnemius muscles (MG and LG), after 3 sets of the 2 minutes performed on the same board [23]. In relation to the AT, two studies investigated the responses to acute stretching. Interestingly, they found a significant AT stiffness increase for either the nondominant limb [24] or the dominant one [21] suggesting that methodological aspects could have contributed to this result and pointing out the need for further studies.

These studies present different methodological approaches, including imaging acquisition protocols and stretching programs. Mostly, the stretching maneuver was very intense and used the dynamometer which is far from the practical routine of daily stretching in therapeutic and physical training interventions. Also, the effect of acute stretching on *µ* for the whole posterior chain muscles has not been studied. The extensibility of the gastrocnemius can influence hip flexion, given its proximal insertion on the posterior region of the knee. When stretching, it may cause discomfort in the gastrocnemius muscles, potentially limiting the individual from maximal stretching of the hamstring muscles [25].

Consequently, it becomes essential to investigate the effect of an acute stretching protocol that includes different exercises for the lower limb posterior chain, with functional exercises that make part of common practical routine. Therefore, this study aimed to investigate the acute responses in the *µ* parameter of the lower limb posterior chain muscles (ST, SM, BF-lh, MG, and LG) and AT before and after one session of static stretching exercises commonly part of clinical or training sessions.

## 2. Methods

This study was approved by the Ethics Committee of the Clementino Fraga Filho University Hospital (HUCFF/UFRJ) (no. 3.672.989). The samples consisted of 15 healthy adults, 8 men and 7 women with mean age: 27.6 ± 5.75 years, weight: 70.91 ± 13.68 kg, and height: 1.71 ± 0.07 cm. The sample was obtained by convenience among students from the Federal University of Rio de Janeiro. The inclusion criteria were young individuals between 20 and 35 years old, who signed the informed consent form and had not participated in stretching programs in the last year. Participants were excluded from the research if they had used anti-inflammatory medication or reported a history of previous surgery or major injury in the lower limbs.

All tests were performed at the nondominant limb, during one visit. The stretching exercises were performed for the triceps surae and for the hamstring muscle groups and the joint ROM measurement (hip or ankle) and the elastographic images (AT, LG, and MG or BF-lh, SM, and ST) were acquired just before and immediately after the exercise for each muscle group.

The beginning of the protocol was randomized among participants, with seven volunteers starting with triceps surae stretching and eight volunteers beginning hamstring exercises. It is important to note that the stretching exercises for each joint remained consistent throughout.

Elastographic images were acquired with an Aixplorer ultrasound system (v.11 Supersonic Image, Aix-en-Provence, France), and a 60 mm linear-array transducer at 4–15 MHz frequency was used for the AT, while a 40 mm linear-array transducer with a frequency of 2–10 MHz was used for the muscles (BF-lh, LG, MG, SM, and ST). Ultrasound gel (Ultrex gel; Farmativa Indústria e Comércio Ltda, Rio de Janeiro, Brazil) was used for acoustic coupling on the surface skin.

**Figure 1:**
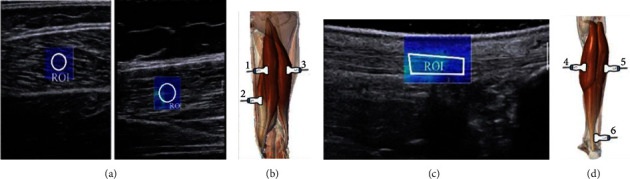
(a) Muscle elastography with ROI positioned in the center of the color map. (b) Measurement sites of ST (1), SM (2), and BF-lh (3). (c) Tendon elastography with rectangle ROI. (d) Measurement site of MG (4), LG (5), and AT (6).

The volunteers lay prone on the stretcher, with their feet hanging relaxed out of the edge, during image acquisition. Reference marks were made at 30% of the leg length (from the popliteal crease to the lateral malleolus) on the MG and LG, according to Lima et al. [17]. With the transducer positioned longitudinally to the fibers, the elastographic mode was activated (Figure 1(d)).

For the hamstring muscles, marks were located longitudinally at 50% (BF-lh and ST) and 75% (SM) of the lower limb length (from the greater trochanter to the lateral epicondyle), as these sites indicate the largest cross-sectional area of the hamstring's muscles [26]. These anatomical points were confirmed by palpation and B-mode images. To distinguish between ST and SM muscles, the inscription of the ST muscle was confirmed. The transducer was placed parallel to the muscle fibers and was held without applying pressure on tissues (Figure 1(b)).

Elastographic images of the AT were acquired with the transducer positioned longitudinally, 2 cm away from its distal insertion, as observed in the B-mode, according to Lima et al. [17]. The transducer was carefully positioned at the skin marks before and after the intervention. For each structure, three elastographic images were acquired.

For data analysis, the images were exported in DICOM format, and the *µ* values were calculated using a custom Matlab R2015a routine (MathWorks, Natick, MA, USA). A circular region of interest (ROI), 1 cm diameter, at the center of the mapping area was used to measure *µ* for the muscles (BF-lh, LG, MG, SM, and ST) (Figure 1(a)). For the AT, a rectangle was selected in the free tendon (Figure 1(c)). The *µ* value was considered the mean of the three images.

The selected exercises are commonly used in practice, and previous studies have demonstrated their effectiveness in significantly increasing ROM after stretching [27, 28]. The subjects participated in a static stretching session for the hamstrings and triceps surae on the nondominant leg. The stretching protocol consisted of four static postures (two for hamstrings and two for triceps sural), with each posture held for 3 × 60 seconds and 30 seconds rest interval for each exercise. Participants were instructed to maintain the maximal joint amplitude, to their individual limit, until the end of each exercise. An adapted pain scale ranging from 0 to 10 was presented to the volunteers, who were instructed to keep approximately an 8 on the scale (adapted from Tibana et al. [29]), where 0 represented the minimum discomfort and 10 the maximum discomfort of pain.

**Figure 2:**
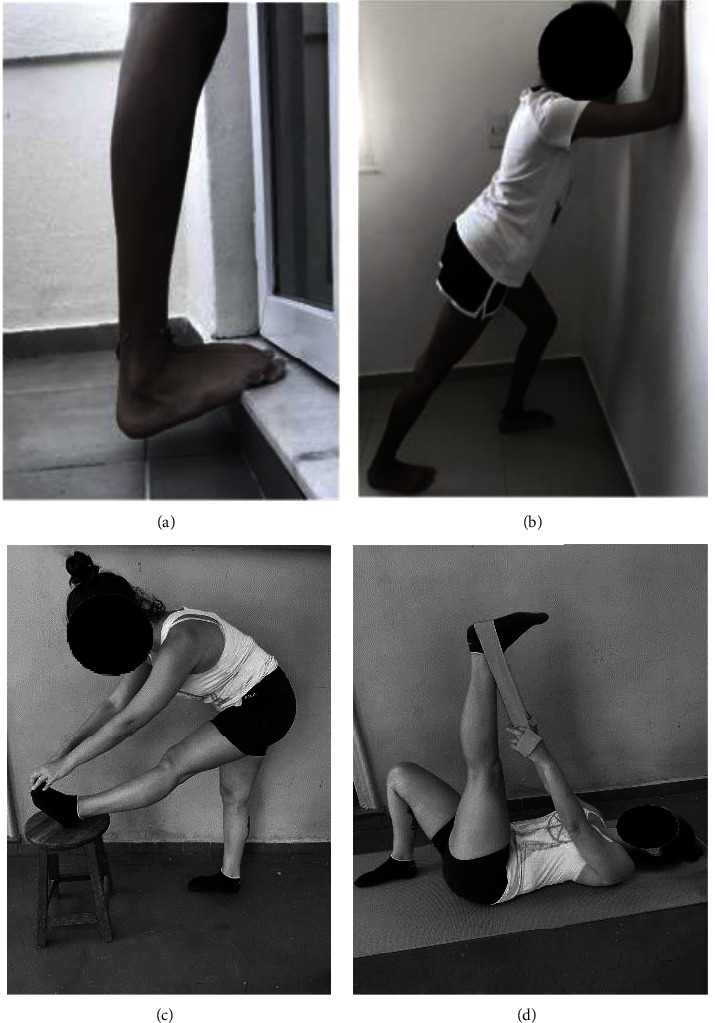
Stretching exercises for (a) triceps surae: heel drop; (b) triceps surae: wall stretch; (c) hamstring: using bench; (d) hamstring: using nonelastic band.

The exercises are listed in Figure 2. For the triceps surae, single-leg heel-drop exercise (Figure 2(a)) and the wall-stretching, without removing the rear foot from the floor (Figure 2(b)). For the hamstrings, trunk flexion with the part of the foot (calcaneus) foot on a 40 cm bench (Figure 2(c)) and hip flexion with the knee extended assisted by a band that does not stretch (Figure 2(d)).

**Figure 3:**
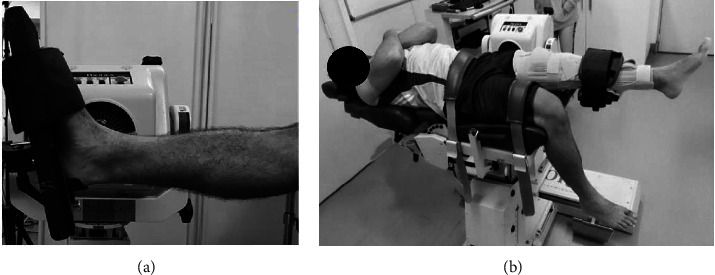
Setup for maximum ROM measurement: (a) dorsiflexion; (b) hip flexion.

To assess the maximum dorsiflexion ROM, the volunteer was positioned seated on the isokinetic dynamometer (Biodex 4 System Pro Medical System Inc., New York, USA) with the knee fully extended and the hip flexed at 85° (0° supine position). The lateral malleolus was aligned with the Biodex's axis of rotation (Figure 3(a)). The test started with 30° of plantar flexion at a constant speed of 5°/s [30]. The participants received exclusively verbal instructions elucidating the test procedures and operational functionality of the equipment. They were then instructed to press a security button to halt the test upon reaching the maximum supported ROM for dorsiflexion or hip flexion. Initially, it was defined that the maximum ROM equated to the highest level of discomfort, designated as a value of 10 on the adapted scale utilized in this study.

To assess the maximum ROM at the hip flexion, the volunteer was placed in a supine position with the nondominant leg attached to the isokinetic dynamometer, and an orthopedic knee immobilizer was used to keep knee extension during hip flexion (Figure 3(b)). The starting position was with the knee and hip joints at 0°. The opposite lower limbs and pelvis were fixed with a dynamometer belt to prevent pelvic tilt due to hamstring extension. The tested hip was passively flexed at a constant velocity of 5°/s, starting from 0° to the final angle, when the participant pressed the button to stop the test at the maximum ROM. For both ROM tests, the amplitude considered was the first trial, with no previous attempts, to ensure the rest state of the tendon.

The intraclass correlation coefficient (ICC) was calculated from the three shear modulus values for all the muscles and the AT, using SPSS 20 (IBM SPSS Statistics Viewer, Armonk, NY, USA). Based on the 95% confident interval, ICC values were interpreted as follows: below 0.49 as poor, 0.5 to 0.75 as moderate, 0.75 to 0.90 as good, and 0.90 to 1.00 as excellent reliability [31].

The Kolmogorov–Smirnov test assessed sample distribution through histogram analysis; a significance level of *p* < 0.05 indicated a nonnormal distribution. The analysis was performed using the Statistica 10 (StatSoft Inc. Tulsa, OK, USA) software. The Kruskal–Wallis test was utilized to analyze categorical factors involving the muscles (BF-lh, ST, SM, LG, and MG) along with the test moments (before and after stretching). The shear modulus (measured in kPa) of the five muscles served as the dependent variable. Post hoc comparisons were performed using Bonferroni's test upon detecting significant effects of *µ* within groups. The analysis was conducted using RStudio version 4.3.2 (RStudio, PBC, Boston, MA). The paired *t*-test was used to compare the before and after stretching values of two variables: ROM, and* µ* of the AT. The significance level for all comparisons was set at 5% (*p* < 0.05). The relative changes in ROM and *µ* for all structures were calculated using ((values after − values before)/values before) *∗* 100. Statistica 10 (StatSoft Inc. Tulsa, OK, USA) was used for the analysis.

In order to identify two subgroups within the sample, the *K*-means classification test was used with two input variables: initial ROM and poststretching ROM variation, for both hip and ankle joints. Additionally, the independent samples test-*t* was used to test the differences in *µ* of muscles and tendon before and after static stretching between the two resulting groups, for those datasets displaying a normal distribution. For variables with a nonnormal distribution, the Mann–Whitney *U* test was used.

## 3. Result

According to the histograms and Kolmogorov–Smirnov tests, it was observed that the data exhibited normality (*p* > 0.05) and homogeneity, except for the following variables: LG after stretching (*p* < 0.001); MG after stretching (*p*=0.004); and ST before stretching (*p*=0.002). As for the intrarater reliability of shear modulus, the ICC ranged from 0.610 to 0.967 for the hamstrings, from 0.911 to 0.996 for gastrocnemius, and from 0.934 to 0.985 for the tendon. These values are considered excellent, except for SM and BF-lh after stretching, which were considered moderate [31].

The maximum ROM and *µ* of muscles and AT are presented as mean ± standard deviation (SD) (Table 1). A significant increase in ROM was observed after acute stretching of hip flexion (ROM HP) and dorsiflexion (ROM DF), with *p* values <0.001.

**Figure 4:**
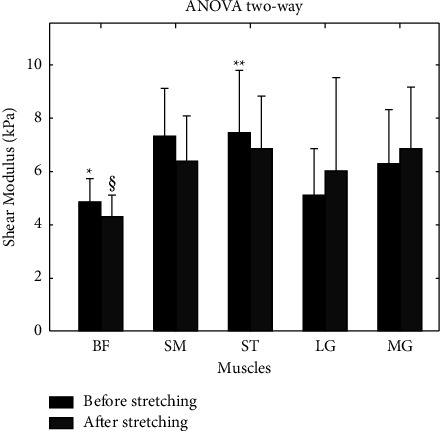
Mean values and standard deviations of each muscle before and after the static stretching session. ^∗^BF-lh before stretching significantly different for ST and SM (before) (*p* < 0.001). ^∗∗^ST before stretching significantly different for LG before (*p* < 0.04). ^§^BF-lh after stretching significantly different for ST, SM, and MG (after) (*p* < 0.001).

Additionally, there were no significant changes in *µ* of the posterior chain muscles after the specific stretching protocol (MG (before-after*p*=0.99); LG (before-after*p*=0.99); AT (before-after*p*=0.14); BF (before-after*p*=0.99); SM (before-after*p*=0.98); and ST (before-after*p*=0.99)). Among the hamstring muscles, the BF-lh showed significantly reduced *µ* values compared to the others (SM and ST), with the same scenario after the protocol (Figure 4).

**Figure 5:**
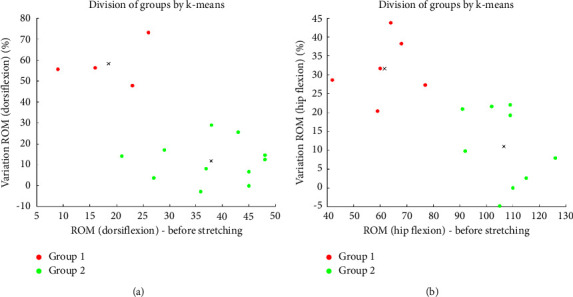
*K*-means clustering analysis of participants based on ROM before stretching and variation ROM after the stretching (Group 1 shown in red and Group 2 shown in green): (a) ankle joint; (b) hip joint.

From the *K*-means, Group 1 includes subjects with lower initial ROM and a greater variation in ROM after static stretching, and Group 2 is formed by individuals with the opposite characteristics (Figure 5(a) for the ankle and Figure 5(b) for the hip).

In the context of the ankle (Figure 5(a)), men and women are equally distributed between groups. Regarding the hip structure, most men are included in Group 1. Interestingly, only 2 male volunteers were placed in the same group (Group 1) for hip and ankle.

Between the two groups, the *t*-test revealed no statistical differences in the stiffness of the triceps sural before and after stretching (before: MG *p*=0.80; LG *p*=0.43; and AT *p*=0.72; after: MG *p*=0.55; LG *p*=0.26; and AT *p*=0.22). For the hamstrings, only the SM showed significantly lower values for Group 2 compared to Group 1 (before: BF *p*=0.25; SM *p*=0.015; and ST *p*=0.67; after: BF *p*=0.78; SM *p*=0.79; and ST *p*=0.84).

## 4. Discussion

The main finding of the present study is that the four stretching exercises commonly applied in the clinical routine did not cause significant changes in *µ* of the muscles of the lower leg posterior chain and of the Achilles tendon. Nevertheless, the intervention was effective in significantly increasing the hip and ankle ROM by about 19.27% and 24.10%, respectively.

The reliability of the shear elastic modulus of muscle and tendon was excellent, except for SM and BF after stretching, considered moderate, in accordance with the literature [31]. The examiner was highly trained, to prevent skin surface compression, which is directly related to changes in the shear modulus [15], and the images were acquired in the longitudinal plane along the longitudinal fibers.

Regarding the hip gain in ROM, these results corroborate other studies, where the hip flexion increases range from 7% to 26%, despite variations in stretching protocol durations, intensities, and exercise types [18–20]. Similarly, for dorsiflexion, the mean relative ROM gain was 7% to 30% [21–23]. A review study indicates that different static stretching protocols targeting hip flexion and dorsiflexion resulted in an average ROM increase of approximately 21% for both joints [31]. Our results for ROM gains were 19% and 24% for the hip flexion and dorsiflexion, respectively, showing that our stretching and ROM test protocols were effective in producing the hip and ankle amplitude gains, based on the literature.

On the other hand, the stretching protocol did not cause acute changes in *µ* of the muscles and of the Achilles tendon. While our study primarily delves into exploring the mechanical characteristics of muscle-tendon units in response to stretching exercises, it is crucial to acknowledge that several factors, beyond muscle and tendon stiffness, contribute to changes in ROM. Recent reviews suggest that changes in sensations like stretch perception and discomfort markedly affect an individual's capacity to endure stretching, consequently impacting ROM [10, 11, 32]. Several studies demonstrate that acute static stretching leads to an augmented range of motion (ROM), reduced passive resistive torque (PRT) [19, 33–36], and increased maximum tolerable PRT [37–39].

Furthermore, other factors contribute to increasing ROM, including neural acute adaptations such as stretch tolerance and alteration in sensory input [7, 8]. These neural factors are under the regulatory influence of the nervous system and are modulated by receptors located in the musculoskeletal structures [40, 41]. Prolonged static stretching (>60 s) is capable of temporarily modifying the H-reflex amplitude, leading to its reduction and subsequently resulting in a reduction of involuntary spinal reflex activity [8]. Those changes in neurophysiology could explain gains in ROM without necessarily reducing muscle or tendon stiffness.

Changes in other nonmuscular structures such as fascia, tendon, and nerves could also explain part of the increased ROM without significant alteration in muscle stiffness [42, 43]. For instance, Andrade et al. [43] investigated acute stretching targeting the sciatic nerve and observed an increase in dorsiflexion ROM with a reduction of nerve stiffness without altering the stiffness of the gastrocnemius muscle. Similarly, fascia and nerve structures can have a strong influence on joint maximal ROM [42].

Previous studies reported a significant decrease in the hamstrings' *µ* after an acute static stretching performed on the isokinetic dynamometer, with durations of 7.5 minutes [18], 5 minutes [20], and 2.5 minutes [19]. Despite the total duration time being similar (6 minutes), our protocol was less intense, resulting in stiffness reductions of about 9.97% for BF-lh, 11.66% for SM, and 6.60% for ST. In comparison, Miyamoto et al. [18] and Nakao et al. [20] found significant stiffness reductions of 27% and 17% for BF-lh, 24% and 30% for SM, and 13% for ST, respectively. The differences in the exercise intensities might account for the variation in the acute response among the hamstring muscles.

For the triceps surae muscles, previous studies report significant reductions in *µ* of the gastrocnemius muscles immediately after 5 minutes of static stretching, either on the stretching board or the wall stretch [21, 22]. Conversely, Akagi and Takahashi [23] did not find a significant difference in the gastrocnemius modulus after 6 minutes of static stretching on the same board, in line with our results.

Although not significant, the relative increase in *µ* for the gastrocnemius in our study was 12.05% for MG and 16.30% for LG. After the stretching, from 15 subjects, 11 showed *µ* increase for MG and 10 for LG. Statistical significance was not reached, based on the large standard deviations. We decided to include two exercises for the ankle stretching protocol, the heel drop, and the wall stretch, as they are currently used in the practical field. The heel drop exercise is commonly applied in clinical settings for the rehabilitation of patients with tendinopathy, particularly due to its eccentric component. When appropriately loaded, it can induce mechanical adaptations in the triceps surae structures, contributing to strength [44]. It is known that after a series of heel drop repetitions, the LG, MG, and AT stiffness increases, depending on the volume, from 11.4% for the MG [44] to 71.7% (LG), 75% (MG), and 41.8% (AT) [44]. The eccentric component of this exercise places a greater load on the muscles and tendon involved, potentially inducing neural responses and reflexes after the application. In our study, the heel drop was applied as a static stretching maneuver, sustained for 180 seconds in total. It is possible that muscle tension might have been intense and induced neural responses, such as reflexes or muscle activity, during the posture. This could explain possible increases in gastrocnemius stiffness for some subjects. This remains an open field for future investigation using electromyography during heel-drop stretching.

There was no significant difference in the AT stiffness after stretching, with a mean relative reduction of about 9.60%. Again, there was a large variability among subjects. A decrease in AT stiffness was reported after acute static stretching with different methods, including ultrasonography and dynamometry [45, 46], whereas others report no change in this property [47, 48]. Surprisingly, the only two studies with SSI showed an increase in AT stiffness after a 5-minute stretching on a stretch board [21, 24]. The possible underlying mechanical or biological mechanisms were not clarified. Another aspect concerns methodological SSI measures of tendons [48]. As stiff and thin structures, the guide-wave phenomenon is possible to occur causing processing errors. As the *µ* calculation involves the tissue density, variations of AT cross-sectional area (CSA) before and after stretching could be another error source. Meanwhile, the biological mechanism that governs the tendon behavior just after removing the stretching tension *in vivo* is still an open topic for research.

Based on biological individuality, it is acceptable that subjects respond at different levels to a given intervention. Categorizing individuals into groups based on peak torque or maximum amplitude after a stretching intervention has already been cited [49, 50]. For a chronic stretching protocol, using the *K*-means classificatory test, some individuals responded to the protocol, increasing the ROM and moving to other groups, while others were considered nonresponders, with no change in classification [51] We identified subgroups by the *K*-means test, based on participants' initial ankle or hip ROM and the amount of variation after the stretching intervention. Two distinct groups were considered: Group 1, characterized by lower initial ROM and greater variation ROM after stretching, and Group 2, mainly comprised of individuals with already higher initial ROM values, exhibiting minimal ROM variation after the intervention, possibly due to their advanced flexibility. It becomes apparent that, for both hip flexion and plantar flexion, the effects of our stretching protocol in joint gaining amplitude seem to be more pronounced in individuals with initial reduced mobility.

There were no statistical differences between the two groups in the stiffness of the gastrocnemius, AT, and hamstrings, except for SM. Before the static stretching, the mean *µ* of SM for Group 1 was significantly higher compared to Group 2. However, after the stretching, no statistical difference was observed between the groups (*p*=0.79). This observation suggested that the higher stiffness in the SM might have contributed to the lower initial ROM. The SM architecture is composed of short and pennate fibers, with tendons overlapping to some extent within the muscle belly [33] which is a design more related to force production than excursion. This may explain the stiffer SM condition of the individuals with less hip joint ROM before stretching. This emerging classificatory approach is relevant for investigating the impacts of stretching, both in acute and chronic contexts, as it highlights the substantial diversity in responses among individuals.

The BF-lh showed less *µ* values than the SM and ST muscles, before and after the static stretching, which can be attributed to variations in muscle anatomy. The SM and ST have overlapping tendinous inscriptions and complex layered networks [33] which could be contributing to greater resting stiffness. In contrast, despite the elongated proximal tendon of the BF-lh, this muscle has a large volume and muscle belly, which may result in lower *µ* values.

There are some limitations in the present study. First, there was a relatively small sample size, with men and women. However, the genders did not present statistical differences for the conditions of amplitude and stiffness of the hamstring and triceps surae structures before and after the static stretching protocol. Second, other structures, such as the hamstring tendons and soleus muscle, were not measured, which limits a comprehensive understanding of the posterior chain behavior in response to stretching. Lastly, the study did not include electromyography (EMG) data to assess muscle activation during stretching, which could have provided additional information about neural responses, if any.

## 5. Perspective

Up to the present moment, it seems that this is the first study that has assessed stiffness changes in the muscles of the leg posterior chain, after a static stretching session, using the elastography SSI technique. Previous investigations have focused on analyzing hamstring or triceps surae muscles. Individuals who practice stretching generally apply the technique to more than one joint. In view of this, the four exercises were applied to two different segments. Although the stretching protocol did not result in significant changes in muscle and tendon stiffness, it demonstrated efficacy in significantly increasing the ROM for the hip and ankle by approximately 19.27% and 24.10%, respectively. These ROM increases align with findings in the literature [18–23]. Notably, individuals with lower initial ROM seem to have better responses to static stretching. These findings suggest that the effects of stretching on ROM are multifactored and extend beyond muscle and tendon properties. Furthermore, it is important to consider that recent studies [10, 11, 32] suggest that alterations in joint range of motion following stretching are influenced by adjustments in individual sensations, such as stretch perception or discomfort, thereby impacting one's tolerance to stretch.

Thus, for the reduction of stiffness in the hamstring musculature to be achieved, previous studies have shown that the most intense stretching protocol, performed on an isokinetic dynamometer, is capable of obtaining these results [18–20]. On the other hand, if there is an increase in the hip joint before the moment of the competition and/or the specific task, this protocol could be sufficient and easy to apply.

The heel drop exercise, commonly used in the rehabilitation of tendinopathy, should be given attention since approximately 66% of the sample showed increases in gastrocnemius stiffness. The observed variations between individuals highlight the importance of considering individual characteristics.

## Figures and Tables

**Table 1:** Range of motion—hip flexion and dorsiflexion.

	Before (mean ± SD)	After (mean ± SD)	Before-after variation (%)
ROM HF (°)	88.61 ± 24.33	103.33 ± 22.68^⸸^	+19.27 ± 13.47
ROM DF (°)	32.73 ± 11.82	38.94 ± 12.09^⸸^	+24.10 ± 22.59

^⸸^Significant difference from before stretching (*p* < 0.001).

## Data Availability

The data used to support the findings of this study are available from the corresponding author upon reasonable request.
